# A systematic review of multi-mode analytics for enhanced plant stress evaluation

**DOI:** 10.3389/fpls.2025.1545025

**Published:** 2025-04-30

**Authors:** Abdolrahim Zandi, Seyedali Hosseinirad, Hossein Kashani Zadeh, Kouhyar Tavakolian, Byoung-Kwan Cho, Fartash Vasefi, Moon S. Kim, Pantea Tavakolian

**Affiliations:** ^1^ Biomedical Engineering Department, College of Engineering and Mines, University of North Dakota, Grand Forks, ND, United States; ^2^ SafetySpect Inc., Grand Forks, ND, United States; ^3^ Mechanical Engineering Department, University of North Dakota, Grand Forks, ND, United States; ^4^ Department of Smart Agricultural Systems, Chungnam National University, Daejeon, Republic of Korea; ^5^ U.S Department of Agriculture/Agricultural Research Service (USDA/ARS) Environmental Microbial and Food Safety Laboratory, Beltsville Agricultural Research Center, Beltsville, MD, United States

**Keywords:** plant stressor, hyperspectral, -mapping indices, stress pattern, multi-modespectroscopy, -stress pathways, -hyperspectral fluorescence imaging (HFI), hyperspectral reflectance imaging (HRI)

## Abstract

**Introduction:**

Detecting plant stress is a critical challenge in agriculture, where early intervention is essential to enhance crop resilience and maximize yield. Conventional single-mode approaches often fail to capture the complex interplay of plant health stressors.

**Methods:**

This review integrates findings from recent advancements in Multi-Mode Analytics (MMA), which employs spectral imaging, image-based phenotyping, and adaptive computational techniques. It integrates machine learning, data fusion, and hyperspectral technologies to improve analytical accuracy and efficiency.

**Results:**

MMA approaches have shown substantial improvements in the accuracy and reliability of early interventions. They outperform traditional methods by effectively capturing complex interactions among various abiotic stressors. Recent research highlights the benefits of MMA in enhancing predictive capabilities, which facilitates the development of timely and effective intervention strategies to boost agricultural productivity.

**Discussion:**

The advantages of MMA over conventional single-mode techniques are significant, particularly in the detection and management of plant stress in challenging environments. Integrating advanced analytical methods supports precision agriculture by enabling proactive responses to stress conditions. These innovations are pivotal for enhancing food security in terrestrial and space agriculture, ensuring sustainability and resilience in food production systems.

## 1 Introduction

Early prediction of plant stress accurately presents complexity due to dynamic interactions among spatial and spectral data, variability in plant responses to environmental stimuli, and limitations in real-time measurements. Furthermore, Improving the tolerance of crop species to abiotic stresses that limit plant growth and productivity is essential for mitigating the emerging problems of global warming (Anshori et al., 2023). Farmers can intuitively detect plant stress events by observing several apparent indicators. For instance, leaf rolling often occurs as plants curl their leaves to conserve water during drought conditions (Kozlowski, 1968). Color changes, such as yellowing leaves, can signal nutrient deficiencies (Marschner and Rengel, 2023), while browning might indicate overwatering or disease (Desai, 2004). Wilting is another clear sign, as drooping leaves or stems suggest insufficient water supply (Jones, 2022). Stunted growth can indicate poor soil conditions or a lack of nutrients, while premature leaf drop often results from environmental stress, such as extreme temperatures. Monitoring a single pattern of stressful events or indicators provides an opportunity to mitigate crop failure.

**Figure 1:**
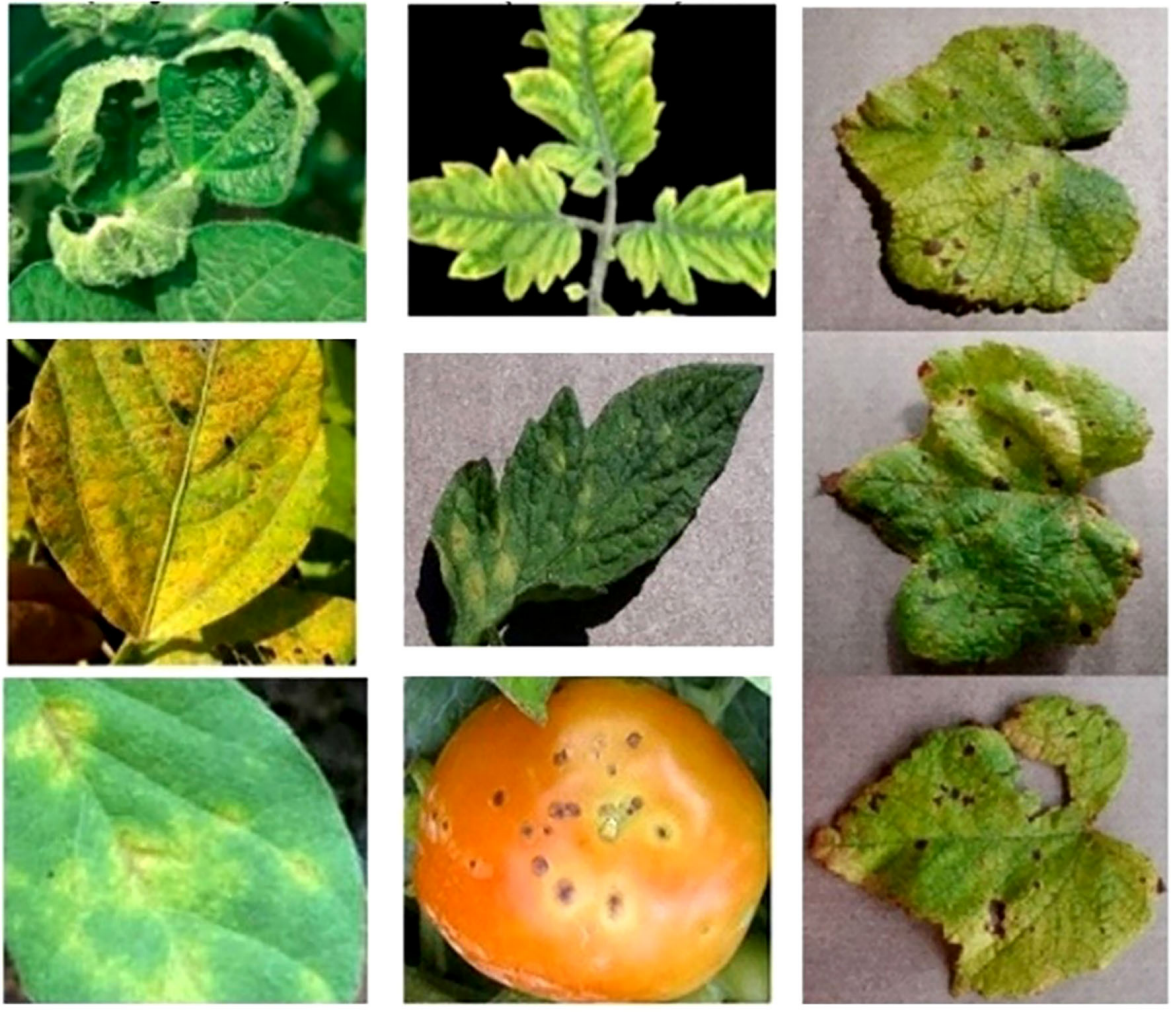
Early stress pattern recognition and mapping the correct indices to the events are crucial for accurate and timely intervention. ([Left column: Bacterial Blight in soybean, top: leaf curling, middle: yellowing, and bottom: structural decay. Shannon, 2021; Hossain, 2023; Tarakanov et al., 2022], [Middle column: Mosaic Virus in tomato, top: stunted and yellow, middle: mosaic appearance, and bottom: Irregular spots. Wolters, 2021; Zhao, 2021; Osdaghi et al., 2021], [Right column: blight disease in grape, appearance and structural change. Yang 2024]).

Multi-mode analytics integrates data from multiple detection modes and spectral bands to model plant stress responses accurately (Coatsworth et al., 2023). It captures real-time data to distinguish transient from prolonged stress while detecting early biochemical shifts in photosynthesis before symptoms appear (Lobos et al., 2021; Wang et al., 2022). Correcting for overlapping spectral signals, such as chlorophyll and non-photosynthetic pigments, enhances accuracy (Miao et al., 2020). Multi-mode systems also track recurrent stress patterns, distinguishing adaptive responses from new stressors and identifying concurrent nutrient and water deficiencies (Li et al., 2024; Iqbal and Munir, 2024). **Figure 1** depicts early biotic stress patterns on apples, cherries, grapes, peaches, peppers, potatoes, squash, and strawberries that can benefit from Multi-mode analytical stress detection.

Single-mode analytics (e.g., UV, IR, Raman) fail to assess multiple stressors simultaneously, limiting plant health insights. Raman spectroscopy detects molecular vibrations but provides limited physiological data. In contrast, multi-mode analytics (MMA) integrates hyperspectral reflectance imaging (HRI), hyperspectral fluorescence imaging (HFI), LiDAR, and Machine Learning (ML) for enhanced stress detection and mapping (Mu et al., 2020). This approach enables near-real-time monitoring of plant responses to biotic and abiotic factors.

—MMA domain includes combinatorial microstate stress patterns, hyperspectral, statistical, and multi-dimensional eigenvector data reduction (Gu et al., 2021) such as Principle Component Analysis (PCA), Partial Least Square Regression, hybrid data fusion, significantly enhance the accuracy of stress indicators, predict the root causes of stress events, expedite data processing, and increase crop yield in terrestrial and space environments.

## 2 Review methodology and strategy

We searched Web of Science (1956 records) and PubMed (418 records) for studies from 2002 to 2024 using keywords related to multimodal plant stress analysis. After removing duplicates, 2,368 records remained. We screened 600 abstracts and selected 350 full-text studies, excluding those focused on single-mode spectroscopy, non-agricultural stress, or non-spectral imaging. The review followed the PRISMA framework for transparency and rigor.

## 3 Plant stress types and responses

### 3.1 Plants respond to stress

Plants respond to stress through various mechanisms and response times. Stress types include abiotic factors like drought, salinity, and heat and biotic factors like pathogens and herbivores (Zhang et al., 2023). Responses such as stomatal closure and antioxidant production can be immediate or delayed, like root elongation and leaf abscission. Mechanisms include morphological changes (e.g., altered root-to-shoot ratio) (Luong and Loik, 2022), physiological adjustments (e.g., osmoregulation), biochemical responses (e.g., antioxidant production, hormone signaling), and molecular changes (e.g., stress-responsive gene expression) (Seok et al., 2023). Outcomes include tolerance through physiological adjustments, avoidance via strategies like stomatal closure, resistance through structural defenses, or escape by completing the life cycle before stress intensifies. Additionally, plants may exhibit hormesis, a beneficial response to low stress levels that enhance growth, development or stress resilience. These adaptations and responses collectively help plants survive and thrive in challenging environments.

In contrast, biotic stresses like pathogenic attacks often manifest over a more extended period, progressing from initial symptoms such as leaf curling to color changes, wilting, and the appearance of lesions or spots. The onset of these symptoms can span several days to weeks, making early detection more challenging. For example, bacterial leaf streaks in rice are caused by Xanthomonas oryzae pv. oryzicola, initiates as thin, water-soaked streaks that gradually turn yellowish-brown, with symptoms developing over several days (Fang et al., 2019). In maize (Zea mays), late wilt disease caused by Magnaporthiopsis maydis develops late in the growing season, with symptoms including rapid wilting and plant death, often after flowering (Degani et al., 2021). These examples underscore the importance of early detection and tailored management strategies for different crops and stress types (Hekimhan and Aydoğdu, 2024). **Table 1** provides classifications of plant stress types and their typical responses ranging from minutes to hours and days.

**Table 1:** Plant stress types and responses.

Classification	Stress Type	Examples	Typical Timing Response (with References)
Based on Stress Type	Abiotic	Drought, heat, cold, salinity, nutrient deficiency, light stress, heavy metals.	Drought stress effects, such as reduced chlorophyll fluorescence and increased NIR reflectance, are detectable within days (Zhang et al., 2023).
	Biotic	Pathogens (bacteria, fungi), herbivores (insects, animals), competition with other plants.	Pathogenic attacks manifest symptoms like leaf curling, yellowing, and lesions over several days to weeks (Yadeta and Thomma, 2013).
Based on Temporal Response	Immediate Responses	Stomatal closure, rapid production of antioxidants, leaf curling.	Occurs within minutes to hours after the stress onset.
	Delayed Responses	Root elongation, leaf abscission, synthesis of protective compounds.	Develops over days to weeks after stress exposure.
Based on Mechanism	Morphological	Increased root-to-shoot ratio, trichome formation, leaf abscission.	Typically observed within weeks, depending on stress severity.
	Physiological	Stomatal regulation, osmoregulation (accumulation of proline, sugars), reduced photosynthesis rate.	Detected within hours to days after stress onset.
	Biochemical	Antioxidant production, hormone signaling (ABA, SA, and JA), secondary metabolites (flavonoids).	Occurs within hours to days, depending on the stress.
	Molecular	Gene expression changes, heat shock proteins, and signaling pathway activation (e.g., MAP kinase).	Gene expression changes can be detected within minutes to hours of stress onset (Von Ziegler et al., 2022).
Based on Outcome	Tolerance	Osmotic adjustment, ROS scavenging, heat shock protein production.	Stress tolerance mechanisms build over days to weeks.
	Avoidance	Stomatal closure, dormancy, altered flowering time.	Avoidance responses can occur within hours to weeks, depending on the strategy.
	Resistance	Structural barriers, production of phytoalexins, jasmonic acid-mediated defense.	Resistance builds over days to weeks after biotic stress exposure.
	Escape	Early flowering production before drought.	Escape mechanisms depend on developmental timing, often weeks to months.
	Hormesis	A Bi-phasic adaptive mechanism occurs when mild stress induces beneficial adaptations. Low levels of stress (e.g., mild drought or light stress) stimulate growth and resilience.	Beneficial hormetic effects can appear within days of mild stress exposure (Kouda and Iki, 2010).

### 3.2 Stress response time

Plants exhibit stress responses that vary in timing and detectability, depending on the stress type and severity. Stress responses depend on environmental variability, sensors, calibration, MMA methods, and sensing devices. Under drought conditions, physiological changes such as reduced chlorophyll concentration can occur within days, detectable through decreased chlorophyll fluorescence in the visible red spectrum and increased leaf reflectance in the near-infrared (NIR) region. For instance, studies have shown that chlorophyll fluorescence parameters can indicate drought stress effects on photosynthesis and secondary metabolism within a week (Zhang et al., 2018). In contrast, biotic stresses like pathogenic attacks often manifest over a more extended period, progressing from initial symptoms such as leaf curling to color changes, wilting, and the appearance of lesions or spots. The onset of these symptoms can span several days to weeks, making early detection more challenging. For example, the progression of vascular wilt diseases involves a series of symptoms that develop over time, complicating timely diagnosis (Yadeta and Thomma, 2013).

### 3.3 Environmental variability

Environmental variability significantly affects the accuracy of MMA methods for plant stress detection and response time assessment. Temperature fluctuations can alter the refractive indices of optical materials, leading to measurement inaccuracies in hyperspectral imaging (Wang et al., 2020). Pressure and humidity changes influence light propagation in multi-mode fibers, affecting the precision of optical coherence tomography (Hyvärinen and Oja, 2000). Moreover, environmental factors modulate plant stress responses, impacting detection times, as observed in crop drought stress studies (Mertens et al., 2023). Advances in electronic technology and software development can enhance the accuracy of MMA, enabling more robust and reliable plant stress detection (Khonina et al., 2023 **).**

### 3.4 Early detection

**Figure 2:**
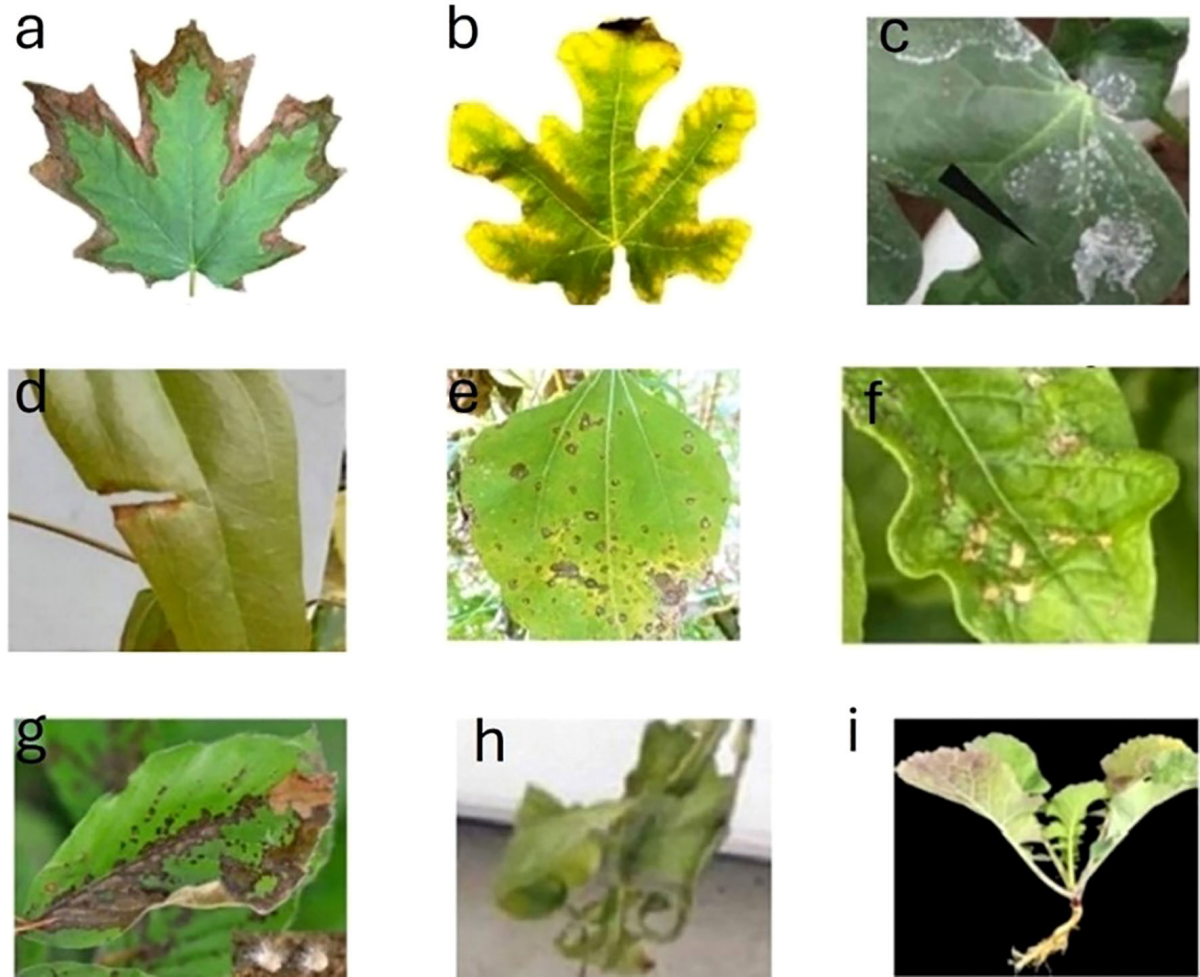
Early detection precedes visible indicators of plant stresses. (**(A)** drought stress [wang, 2023], **(B)** nutrient deficiency [Garza-Alonso et al., 2019], **(C)** salinity [Peng et al., 2016], **(D)** heat stress [Bi et al., 2020], **(E)** pathogen attack [Shafique et al., 2022], **(F)** pest infection [Li et al., 2024], **(G)** fungal infection [Gossner et al., 2021], **(H)** cold stress [Primo-Capella et al., 2021], and **(I)** waterlogging [Hong et al., 2024]).

**Figure 3:**
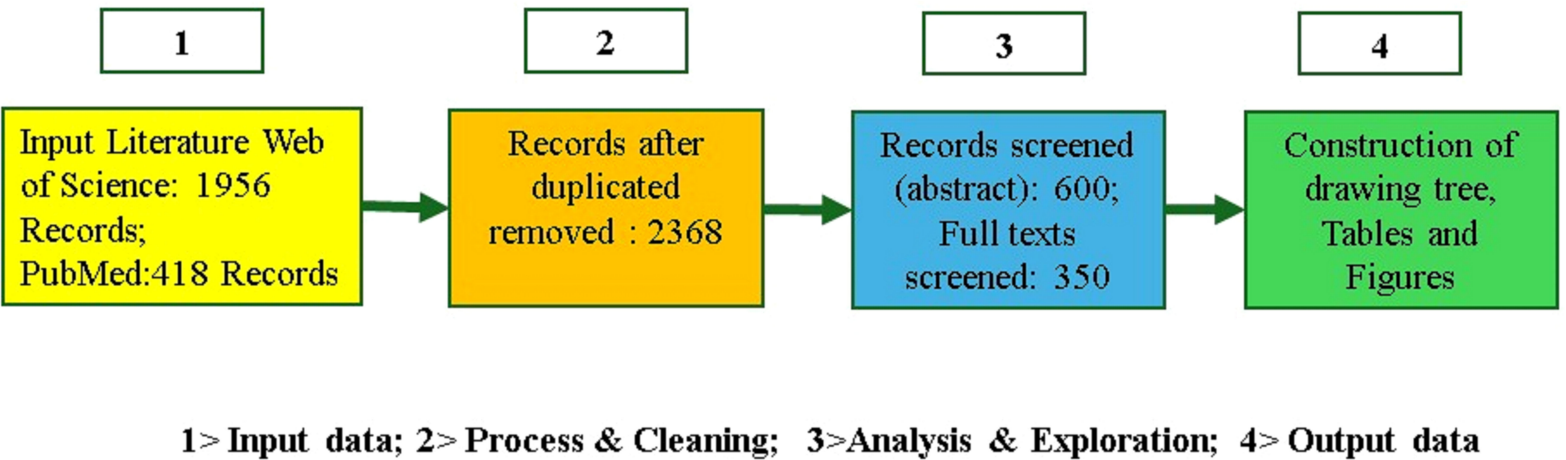
Block diagram of the systematic approach used to review the topic of multi-mode analytics.

The key features of early stress detection include detecting non-visible indicators such as altered chlorophyll fluorescence emission, leaf reflectance/absorption, or thermal signature, often observable within days of stress onset (Zhang et al., 2018). **Figure 2** depicts some of the visible indicators of plant stresses. Early detection precedes signs of stress and enables preventive actions like adjusting irrigation or applying treatments to avoid long-term damage. Stress-specific patterns, such as drought-induced photosynthesis reductions or pathogenic attacks’ sequential symptoms, require tailored detection approaches. By preserving plant health, minimizing resource use, and improving precision agriculture, early detection is critical for optimizing productivity (Yadeta and Thomma, 2013). **Figure 3** provides examples of various types of plant stress. Early detection means predicting plant stress before visible signs of stress appear.

## 4 Value of multi-mode analytics

Analytics systematically analyzes plant health, growth, and stress data to support informed agricultural decisions (Kamilaris et al., 2017). This process collects and interprets data from sensors, satellite imagery, and laboratory tests to identify patterns that guide management practices (Sarfraz et al., 2023). It enables early stress detection, allowing timely interventions (Aina et al., 2024). Lenk et al. (2007) used multispectral fluorescence and reflectance imaging to assess leaf-level stress responses, identifying fluorescence signatures linked to chlorophyll and other pigments. Lang et al. (1996) demonstrated that fluorescence imaging detects physiological changes by analyzing red and far-red chlorophyll fluorescence under drought stress. Leufen et al. (2014) combined fluorescence indices and reflectance data to differentiate abiotic and biotic stressors, enhancing stress monitoring precision.

The shift from single-mode to multi-mode approaches has improved plant stress detection by integrating multiple spectral bands and detection modes. Thermal imaging enhances water stress monitoring in almonds, aiding targeted irrigation (García-Tejero et al., 2018). Hyperspectral and thermal imaging combined improve drought stress and transpiration assessments (Mertens et al., 2023). Chlorophyll fluorescence detects phosphorus deficiency (Frydenvang et al., 2015), while AI-assisted fluorescence identifies nutrient deficiencies (Aleksandrov, 2019). Key multi-mode analytics features include stress pattern recognition and indicator mapping.

-One of the critical features of the multimodal analytical approach is stress pattern recognition and stress indicator mapping, which enables early detection and differentiation of biotic and abiotic stressors (Fiorani and Schurr, 2013). Additionally, it integrates diverse detection modes—such as hyperspectral reflectance, fluorescence, and thermal imaging—to capture complementary information about plant physiology (Mertens et al., 2023). This approach enhances the sensitivity and specificity of stress detection by leveraging data from multiple spectral bands (e.g., VIS, NIR, SWIR) and combining optical and non-optical datasets. Multimodal analytics also supports advanced ML techniques for refining analysis and automating processes while addressing practical challenges like calibration, data management, and cost efficiency (Fiorani and Schurr, 2013; Jones et al., 2019). Multi-mode Analytics increases the possibility of Predicting the Root Cause of plant stress. Cross-modal contribution is another essential feature unavailable in single-mode analytics, which leads to monitoring errors.

### 4.1 Mapping and pattern recognition

Pattern recognition identifies the sequence of stress indicators, while mapping quantifies their severity and distribution. For instance, a typical water stress pattern might involve initial leaf edge curling, followed by an increase in near-infrared reflectance as seen in the Normalized Difference Vegetation Index (NDVI), and a subsequent decrease in Chlorophyll Content Index (CCI) (Zubler and Yoon, 2020). Mapping these indicators allows researchers to develop a framework for early water stress detection, which can be vital in environments where resource limitations or mission constraints make rapid interventions necessary (Farella et al., 2022).

### 4.2 Integration

MMA enhances plant stress detection by integrating diverse sensing methods. Hyperspectral Fluorescence Imaging (HFI) captures chlorophyll fluorescence, revealing early stress indicators (Moustaka and Moustakas, 2023). Hyperspectral Reflectance Imaging (HRI) assesses pigment concentrations and water content, while thermal imaging detects canopy temperature changes linked to water stress. LiDAR provides structural data, improving the interpretation of reflectance and fluorescence signals (Meemken, Becker-Reshef, et al., 2024). This synergy enhances sensitivity and specificity, linking physiological responses to structural changes for comprehensive plant monitoring.

### 4.3 Enhanced sensitivity and specificity

Multi-HRI offers detailed spectral information by capturing a wide range of wavelengths that provide insights into various aspects of plant physiology, including the health of plant pigments, water content, and cellular structure (Maimaitijiang et al., 2020). This capability identifies subtle changes that might indicate stress before visible symptoms become apparent.

### 4.4 Predicting plant stress root causes

Predicting plant stress root causes relies on ML and multimodal data fusion (MMDF) to analyze complex datasets and identify hidden patterns (Li & Zhao, 2022). MMDF integrates diverse data streams for clearer insights (Bokade et al., 2021). For instance, correlating NDVI with the Red-Edge Inflection Point (REIP) helps detect water stress early by linking chlorophyll loss to canopy impairment (Arief et al., 2023). Similarly, combining the LiDAR-derived Leaf Area Index (LAI) with the Photochemical Reflectance Index (PRI) reveals nitrogen deficiency patterns (Lefsky et al., 2002). Such integrations enhance early and precise stress detection.

### 4.5 Cross-modal contribution

Chappelle et al. (1992) determined that the contribution of chlorophyll fluorescence in the Red-NIR (680-750 nm) region of the reflectance spectra can be as great as 23% of reflectance at 685 nm and 4% at 740 nm. for soybeans and fluorescence changes in reflectance have minimal contribution to the “red edge shift” effect. Kim et al. (1993) developed the Ratio Analysis of Reflectance Spectra (RARS) algorithm to estimate chlorophyll a, b, and carotenoid concentrations in plant leaves. This process amplifies the absorption band maxima and minima specific to each pigment. By comparing the ratio spectrum with the absorption spectra of pure pigments, they identified inflection points related to chlorophylls and carotenoids. The RARS algorithm uses these ratio spectra to establish linear solid relationships between absorption bands and pigment concentrations, forming the foundation for accurate equations to estimate these pigment levels in leaves.

## 5 Photon-leaf interaction

Within the visible spectrum (400-700 nm), chlorophyll absorbs blue and red light for photosynthesis, while reflected green light gives leaves their color (Gitelson et al., 2020). Some absorbed energy is re-emitted as fluorescence, but most drives chemical reactions. In the NIR region (0.7-1.3 µm), absorption decreases, and internal leaf structures enhance reflectance, regulating leaf temperature (Jacquemoud and Ustin, 2001). In the SWIR range (1.3-2.5 µm), water and biochemical compounds absorb photons, reducing reflectance and revealing hydration status and biochemical composition (Ceccato et al., 2001).

### 5.1 Visible spectrum (VIS: 400-700 nm)

Red, Green, and Blue Reflectance (RGB) offer insights into plant health. Reflectance in the red band (620-750 nm) assesses photosynthetic activity because chlorophyll absorbs this band heavily (Tucker, 1979). High reflectance in the green band (495-570 nm) typically indicates a healthy, chlorophyll-rich plant, while low reflectance may suggest stress or disease (Huete et al., 2002). The blue band (450-495 nm) also relates to chlorophyll absorption and is an additional plant health indicator. Variations in these reflectance values are often early indicators of stress before visual symptoms appear, making VIS a valuable tool for plant monitoring (Elvidge and Chen, 1995).

### 5.2 NIR (0.7-1.1 µm)

NIR (0.7-1.1 µm) reflects plant structure and water content, aiding biomass and health estimation (Pohl and Van Genderen, 2016). Red-edge reflectance (680-750 nm) is sensitive to chlorophyll changes and stress (Krezhova, 2011). High NIR reflectance signals healthy vegetation, while declines indicate stress (Keshava and Mustard, 2002). NIR fluorescence detects secondary metabolites and structural changes, revealing stress responses beyond the visible spectrum (Kumar et al., 2024).

### 5.3 Shortwave infrared (SWIR: 1.1–2.5 µm)

The spectrum is essential for assessing plant water content and structural attributes. Reflectance in this region decreases with increased leaf water content, making it a reliable indicator for detecting drought stress and optimizing irrigation (Ceccato et al., 2001). Additionally, SWIR bands are sensitive to plant biochemical properties, including lignin and cellulose, providing insights into plant structure and biomass (Asner, 1998). These features make SWIR crucial for understanding water use efficiency and evaluating plant resilience under environmental stress.

### 5.4 Longwave infrared (LWIR: 8–14 µm)

The Longwave Infrared (LWIR) spectrum focuses on thermal emissions and canopy temperature directly linked to plant transpiration and water stress. Elevated canopy temperatures, observed through LWIR, often signal reduced transpiration due to water stress or stomatal closure, enabling early detection and precise irrigation scheduling (Jones et al., 2009). Furthermore, LWIR data can identify thermal anomalies caused by plant diseases or pest infestations, offering a non-invasive diagnostic tool for plant health (Oerke et al., 2014). These capabilities make LWIR indispensable for monitoring plant-environment interactions and managing stress-related impacts effectively. **Table 2** lists the Reflectance detection mode and their function across multiple spectral bands.

**Table 2:** Reflectance detection mode across multiple spectral bands.

SPECTRAL RANGE	FUNCTION (*)	REFERENCES
Visible Spectrum (VIS: 400-700 nm)	(↑) Red Reflectance (620-750 nm): indicates lower chlorophyll content. (↑) Green Reflectance (495-570 nm): suggests a healthy, chlorophyll-rich plant; (↓) reflectance may indicate stress or disease. (↑)(↓) Blue Reflectance (450-495 nm): This is related to chlorophyll absorption and is an additional plant health indicator. (↓) Chlorophyll Fluorescence (around 685 nm and 740 nm): signals stress from water scarcity or nutrient deficiencies.	Lichtenthaler et al., 2005
Near-Infrared (NIR: 7-1.100 µm)	(↑) NIR Reflectance 700-1100 nm: Healthy vegetation, such as healthy plants, reflects more NIR light due to its dense cellular composition. (↓) Red-Edge Reflectance (680-750 nm): can signify plant stress, often due to water deficit or disease. Susceptible to changes in chlorophyll concentration (↑)(↓) NIR Fluorescence (700-800 nm): Can reveal leaf structure or internal water content alterations indicative of plant health.	Gitelson et al., 1994; Blackford, 2010
Short-Wave Infrared (SWIR: 1.1-2.5 nm µm)	(↑)(↓) SWIR Reflectance 1000-2500 nm: Assess water content and biochemical composition in plants, including substances like lignin and cellulose (↑) SWIR Fluorescence: May indicate stress-related changes in cell wall structure or biochemical composition.	Jackson et al., 2022
Thermal Infrared (TIR: 8-14 µm) LWIR	TIR Emission: Elevated temperatures often indicate plant stress, particularly from water deficit or reduced transpiration. TIR data is critical for assessing plant stress, especially under environmental Stress conditions.	Mertens et al., 2023
LiDAR (0.7 -1.55 µm)	LiDAR (700-1550 nm) (↑) Canopy Height: Increases in canopy height indicate healthy growth and structural integrity. (↓) Leaf Area Index (LAI): Decreases in LAI may signal plant stress or reduced growth. (↑)(↓) Leaf Tilt Index (LTI): Changes in leaf orientation can reveal adaptive responses to stressors such as light availability or water deficit.	Lefsky et al., 2002

* (↓), (Decreased Reflectance; (↑), Increased Reflectance).

## 6 Stress Indicators In Different Spectral Bands

### 6.1 Hyperspectral fluorescence imaging

Indices (**Table 3**) correlate fluorescence emission of chlorophyll or Xanthophyl within one or more spectral bands. **Table 3** provides a partial list of indices related to chlorophyll pigments in a plant. Each index quantifies certain aspects of plant health. Chlorophyll a (Chl a) converts light energy into chemical energy and plays a role in the Calvin cycle and redox signaling pathways during stress (Foyer and Shigeoka, 2011). Chlorophyll b (Chl b) maximizes light absorption and adjusts to varying light conditions (Bennett, 1983). Beta-carotene acts as an antioxidant, stabilizing membranes and protecting against oxidative stress. Xanthophylls are involved in photoprotection and regulating energy dissipation (Quaas et al., 2015). Chl a serves as the primary pigment for converting light energy into chemical energy during photosynthesis, while Chl b works in tandem to widen the absorption spectrum (Bennett, 1983).

**Table 3:** Stress indices due to HFI on chlorophyll pigments.

LABEL	STRESS INDEX	EXPLANATION
Chlorophyll Fluorescence Ratio	$\text{CFR}=\frac{F_{685}}{F_{740}}$ ​​	Measures the ratio of fluorescence emissions at 685 nm and 740 nm to assess photosynthetic efficiency.
Photochemical Reflectance Index	$\text{PRI}=\frac{F_{531}-F_{570}}{F_{531}+F_{570}}$	Carotenoid responds to light stress, calculated from 531 nm and 570 nm fluorescence emissions.
Chlorophyll Content Index	$\text{CCI}=\frac{F_{750}-F_{705}}{F_{750}+F_{705}}$	Estimates chlorophyll content using emissions at 750 nm and 705 nm to reflect plant health.
Xanthophyll Index	$\text{XI}=\;\frac{F_{550}-F_{520}}{F_{550}+F_{520}}$	To estimate xanthophyll activity by comparing fluorescence emissions at 550 nm and 520 nm, indicating stress responses and photoprotection.
$F_{v}/F_{m}$ Ratio (Maxwell and Johnson, 2000)	$Fv/F_{m}=\frac{F_{v}}{F_{m}}$	Indicate photosystem II efficiency and overall plant health.
Non-photochemical quenching) (Demmig-Adams, 1990)	$\text{NPQ}=\frac{F_{m}-F_{m}^{"}}{F_{m}^{"}}$ ​​	Measure the proportion of light absorbed by chlorophyll, dissipated as heat. F_m_: Maximum fluorescence yield of a dark-adapted sample. F_m_’: Maximum fluorescence yield of a light-adapted sample.
Fluorescence Index (FI) (Zarco-Tejada et al., 2012)	$FI=\frac{F_{690}}{F_{520}}$	Quantify plant health by comparing fluorescence at specific bands.

### 6.2 Hyperspectral reflectance imaging

Hyperspectral Reflectance Imaging (HRI) indices track plant stress by analyzing changes in absorption and reflectance patterns. Reduced chlorophyll levels alter these patterns, signaling stress. Water in leaves absorbs light in the Shortwave Infrared (SWIR) region (1.1–2.5 µm), making SWIR crucial for detecting dehydration and drought stress (Ceccato et al., 2001). Leaf cellular structure, including mesophyll thickness and air spaces, scatters light in the Near Infrared (NIR) region (0.7–1.3 µm), where minimal absorption enhances reflectance, revealing biomass and structural integrity (Jacquemoud et al., 2009). Non-pigment components like lignin and cellulose influence SWIR reflectance, providing insights into plant structure and maturity. **Table 4** Lists HRI modes, indices, and their corresponding stress analysis functions.

**Table 4:** HRI indices provide pathways for stress analysis.

MODE	LABEL	STRESS INDEX [Note]	PURPOSE
Reflectance (VIS)	Normalized Difference Vegetation Index (NDVI)	$NDVI=\frac{\left(NIR-RED\right)}{\left(NIR+RED\right)}$	Assesses vegetation health by comparing near-infrared and red reflectance (Rouse et al., 1974)
	Visible Atmosphere Resistant Index (VARI)	$VARI=\frac{\left(GREEN-RED\right)}{\left(GREEN+RED-BLUE\right)}$	Evaluates vegetation health while minimizing atmospheric effects. (Gitelson et al., 2003)
	Chlorophyll Content Index (CCI)	$CCI=\frac{\left(RED-BLUE\right)}{\left(RED+BLUE\right)}$	Estimates chlorophyll content based on visible spectrum reflectance. (Gitelson et al., 2003)
	Normalized Difference Water Index (NDWI)	$NDWI=\frac{\left(NIR-SWIR\right)}{\left(NIR+SWIR\right)}$	Measures plant water content by comparing NIR and SWIR reflectance (Gao, 1996)
	Normalized Difference Vegetation Index	$NDVI=\frac{\left(NIR-RED\right)}{\left(NIR+RED\right)}$	General vegetation health and biomass estimation. (Rouse et al., 1974)
Reflectance (SWIR)	Normalized Difference Water Index	$NDWI=\frac{\left(NIR-SWIR\right)}{\left(NIR+SWIR\right)}$	To evaluate vegetation water content. (Gao, 1996)
Reflectance (Red-Edge)	Red-Edge Vegetation Index (REVI)	$REVI=\frac{\left(NIR-RED_{edge}\right)}{\left(NIR+RED_{edge}\right)}$	Quantify sensitivity to chlorophyll content and plant health in the red-edge region (Krezhova, 2011)
Reflectance (Red-Edge)	Red-Edge Chlorophyll Index (CIred-edge)	$CI_{red-edge}=\frac{NIR}{RED_{edge}}-1$	Focuses on chlorophyll content by comparing reflectance in NIR to red-edge reflectance (Gitelson et al., 2003)

### 6.3 Red edge shift in the VIS band

**Figure 4:**
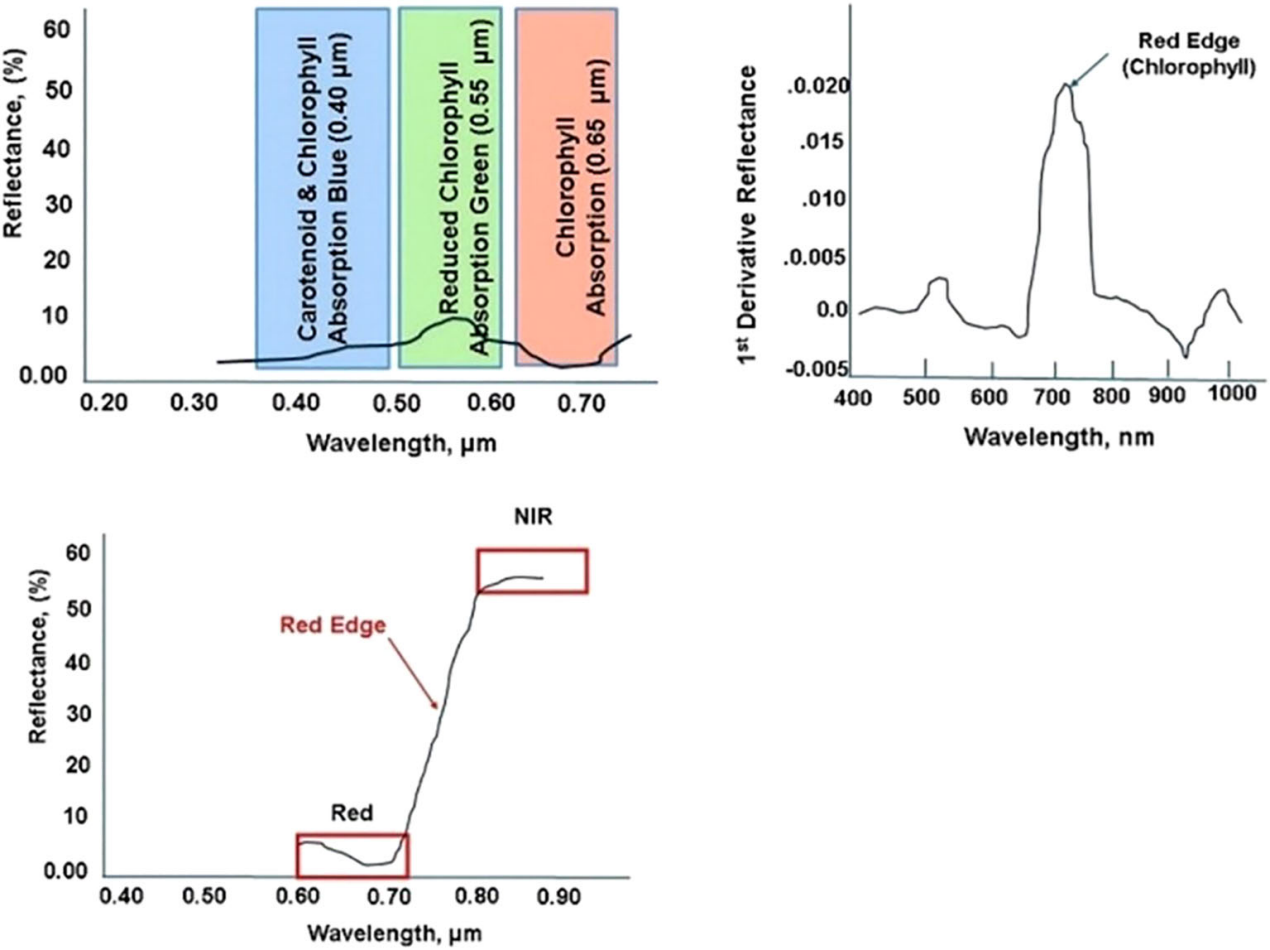
Stress within Visual (Blue, Green, Red) bands (upper left); Red Edge transition within Visible Near Infrared bands (lower middle); Red Edge (upper right) first derivative to enhance detection sensitivity (Horler et al., 1986; Filella et al., 1992).

Red Edge Shift in the VIS Band (**Figure 4**) refers to the alteration in the wavelength of the peak reflectance in the red-edge region of the electromagnetic spectrum (approximately 680-750 nm). Red Edge detects vegetative stress earlier in the plant growth cycle. As plants experience stress, chlorophyll concentration often declines, leading to decreased light absorption in the red region (around 680 nm) and increased reflectance at longer wavelengths. The shift toward the infrared part of the spectrum (700 nm and beyond) indicates changes in plant health, biochemical composition, and physiological status (Tucker, 1979). The first derivative of the reflectance spectrum enhances the detection of subtle shifts by highlighting peaks and inflection points, making it easier to observe changes in plant health and stress responses.

### 6.4 Longwave infrared, LWIR (8 -14 µm)

LWIT measures plant thermal emission to assess canopy and leaf temperatures. Elevated temperatures indicate stress conditions, such as reduced transpiration due to water deficit (Farella et al., 2022). TIR can identify specific areas of a crop affected by drought stress, enabling targeted irrigation practices that optimize water use efficiency by revealing localized heat patterns. **Table 5** depicts standard thermal and LIDAR indices.

**Table 5:** LWIR and LiDAR indices.

Mode	Label	Stress Indicator [Note]	Purpose
LIDAR	LIDAR Height Index, H	H= Height	To Assess plant height and canopy structure. Direct measurement of plant height (Lefsky et al., 2002)
	LIDAR Leaf Area Index (LAI)	$\text{LAI}=\frac{\text{Total Area}}{\text{Ground Area}}$	Estimates leaf area index to assess plant growth and biomass. Based on canopy height and density metrics (Lefsky et al., 1999)
	LIDAR Leaf Tilt Index (LTI)	$\text{LTI}=\frac{{\text{F}}_{\text{Leaf}}-{\text{F}}_{\text{Reference}}}{{\text{F}}_{\text{Leaf}}+{\text{F}}_{\text{Reference}}}$	Calculates the tilt of leaves to correct distortion in reflectance measurements.; ${\text{F}}_{\text{Leaf}}\;$ = fluorescence emitted by the leaf, ${\text{F}}_{\text{Reference}}\;$ = fluorescence emitted by a Reference sample (Farooq et al., 2020)
Thermal (TIR)	Vegetation Temperature Condition Index (VTCI)	$\text{VTCI}=\frac{\left(\text{LST}-{\text{LST}}_{\text{min}}\right)}{\left({\text{LST}}_{\text{max}}-{\text{LST}}_{\text{min}}\right)}$	To evaluate plant stress based on thermal emissions (Wan, 2002). LST = Land Surface Temperature,

### 6.5 LIDAR (0.7–1.55 µm)

LIDAR (0.7–1.55 µm) uses NIR laser pulses to generate high-resolution 3D plant data by measuring pulse return times. This method estimates key parameters like Leaf Area Index (LAI) and Leaf Tilt Index (LTI), which indicate plant health and productivity. LAI, representing leaf area per ground unit, reflects photosynthetic capacity, influencing growth, transpiration, and soil moisture dynamics (Chen and Cihlar, 1995). It regulates energy balance, affecting absorption, reflection, and climate modeling. LTI quantifies leaf orientation, refining photosynthetic modeling and improving water use efficiency and stress response estimations (Hopkinson et al., 2013).

## 7 Multi-mode analytical methods

Multi-mode analytical methods integrate diverse tools like Dimensionality Reduction (e.g., PCA, PLSR) to extract meaningful patterns from complex datasets. Combining hyperspectral analysis, data fusion, and ML techniques enhances stress detection by identifying subtle changes in plant health. These methods detect stressful events earlier, recognize stress patterns, and provide actionable insights for precision agriculture and ecosystem management, improving plant resilience and productivity.

### 7.1 Dimensionality reduction

Dimensionality Reduction (**Table 6**) simplifies datasets by reducing variables while preserving essential information. These methods identify maximum variation in stress indicators, aiding in trend detection and mitigating overfitting. Many rely on eigenvalues and eigenvectors to transform data into a new space. PCA captures variance through principal components (Van der Maaten et al., 2009), while LDA maximizes class separability (Lu et al., 2011). PLSR models latent variable relationships, and CCA uncovers dataset correlations. ICA separates mixed signals (Hyvärinen and Oja, 2000), while nonlinear methods like t-SNE and UMAP maintain neighborhood structures for visualization.

**Table 6:** Classification of dimensionality reduction methods.

Classification	Description
Principal Component Analysis	It uses eigenvalues and eigenvectors to identify directions of maximum variance.
Linear Discriminant Analysis	Maximizes class separability by finding linear combinations of features.
Partial Least Squares Regression	Identifies latent variables that explain variance in independent and dependent variables.
Canonical Correlation Analysis	Uncovers relationships between two datasets using eigendecomposition.
Independent Component Analysis	Separates mixed signals into statistically independent components.
t-SNE	Preserves neighborhood structures for data visualization in nonlinear relationships.
UMAP	Captures complex nonlinear relationships for visualization or analysis.

### 7.2 Data fusion

Data Fusion (**Table 7**) highlights data fusion as a method for integrating spatial, spectral, and temporal data to enhance plant stress analysis. Spatial data from LiDAR captures canopy structure, including leaf orientation and density, influencing light interception (Berger et al., 2022). Spectral data from hyperspectral imaging detects biochemical changes, such as pigment and water content variations. Temporal data from repeated measurements tracks stress progression and recovery. ML models integrate these dimensions, linking spectral reflectance (chlorophyll content) with spatial anomalies (canopy gaps) and temporal trends (drought stress), improving detection accuracy (Sagi et al., 2020).

**Table 7:** Data fusion techniques in plant stress analysis.

Aspect	Description	Examples
Data Sources	Combines multiple sensing modalities for stress analysis. (Berger et al., 2022)	Hyperspectral imaging, thermal infrared, LiDAR, chlorophyll fluorescence, and soil moisture sensors.
Fusion Techniques	Integrates data at different levels to enhance insights. (Sagi et al., 2020)	Feature-Level Fusion: Combines spectral indices and temperature data. Decision-Level Fusion: Analyzes and then integrates results from different sensors.
Machine Learning	Utilizes advanced models for processing and analysis of fused datasets. (Meng et al., 2020)	Random Forests, Artificial Neural Networks, Support Vector Machines.
Spectral and Temporal Fusion	Fuses data from different spectral bands over time for comprehensive analysis. (Gunatilaka and Baertlein, 2001; Shen et al., 2016)	Spectral Fusion: Integrates visible, NIR, and SWIR bands. Temporal Fusion: Monitors stress progression across time.

### 7.3 Variability in spectral data interpretation

Variability in spectral data interpretation across different environments arises from atmospheric conditions, soil composition, and plant phenotypes, affecting measurement consistency. Differences in light intensity and angle influence reflectance values, requiring adaptive (dynamic correction) calibration techniques to ensure accuracy (Xie et al., 2023). Additionally, spectral shifts due to environmental stressors necessitate robust ML/AI models to account for regional variations and improve data reliability (Hilbert, 2016). MMA devices address these challenges by integrating multiple spectral bands, combining reflectance, fluorescence, and LiDAR data to cross-validate results, and applying AI-driven correction algorithms to enhance consistency across diverse conditions.

Levels of Data Fusion. Data fusion is typically categorized into three levels: low-level, mid-level, and high-level fusion, each defined by the stage at which data integration occurs and the type of information processed (**Table 8**).

**Table 8:** Data fusion levels.

Fusion Level	Description	Example	Reference
Low-Level Fusion	Combines raw data from multiple sensors to produce new raw data, enhancing the quality and completeness of the information.	Merging data from multiple cameras to improve image resolution.	Klein, 2004
Mid-Level Fusion	Integrates features or patterns extracted from raw data to interpret meaningful characteristics from multiple sources.	Combining spectral and textural features from remote sensing data for land cover classification.	Smolinska et al., 2019
High-Level Fusion	Combines decisions or interpretations from multiple sources to make final decisions or predictions by integrating outputs from multiple systems.	Merging diagnostic results from different medical imaging systems.	Hall and Llinas, 1997

### 7.4 Hyperspectral imaging

The hyperspectral analysis represents a crucial subset of multi-mode analysis, distinguished by its comprehensive examination of plant health through essential elements: spectral, spatial, and temporal. Integrating ML algorithms with image data sets enables precise spectral-spatial-temporal disease identification, facilitating timely detection, predictive modeling, and effective disease management (Razzaq et al., 2024). The term “hyperspectral” derives from the method’s ability to capture and analyze many contiguous spectral bands across various wavelengths, allowing for detailed characterization of plant responses to multiple stressors (Gao et al., 2020).

**Spectral analysis** enhances plant stress detection through specific spectral indices, unmixing methods, and matching techniques. The NDVI is well-known for its effectiveness in assessing overall vegetation health and stress levels by exploiting the differences between near-infrared and red reflectance (Rouse et al., 1974). The PRI is sensitive to changes in carotenoid pigments linked to photosynthetic stress, while the Red-Edge Position (REP) indicates shifts in chlorophyll content and health (Gitelson et al., 2003; Zarco-Tejada et al., 2012). Spectral unmixing methods like the Fractional Cover Index (FCI) estimate the proportions of varying vegetation components within pixels. At the same time, the Spectral Angle Mapper (SAM) score quantifies the similarity between pixel and reference spectra to highlight specific stress indicators (Lowe et al., 2017).

**Temporal analysis** monitors plant health and detects stress by evaluating changes in spectral data over time, mainly about specific phenological events. This method employs time-series indices such as the Vegetation Condition Index (VCI) and the Normalized Difference Moisture Index to track vegetation’s dynamic responses to environmental changes and stressors. For example, VCI compares current NDVI values against historical data to assess relative stress levels and identify stress onset and progression (Friedl and Brodley, 1997).

**Spatial analysis** examines the spatial patterns and relationships within remote sensing images. Techniques like Object-Based Image Analysis allow researchers to segment imagery into meaningful objects, such as leaves or plant canopies, to analyze their spatial characteristics and assess vegetation health (Kureel et al., 2022). The VFI quantifies the proportion of vegetation within image segments, enabling accurate assessments of vegetation cover and identification of stress areas (Pettorelli et al., 2018).

## 8 Machine learning

ML is a subset of AI that enables computer systems to learn from data and improve their performance on specific tasks without explicit programming. This approach utilizes algorithms and statistical models to identify patterns within data, allowing systems to make predictions or decisions based on new input (Ratner, 2017). Shahoveisi et al. (2023) reported using image processing and transfer learning for rust detection, evaluating the performance of four different pre-trained CNN models: Xception, ResNet50, EfficientNetB4, and MobileNet. Furthermore, adaptive phenotyping, such as IBP, can utilize ML/AI to integrate high-throughput imaging, automation, and data analytics to assess plant stress responses across growth stages (Anshori et al., 2023).

### 8.1 Edge computing

Edge computing processes data at the network’s edge, enabling real-time analysis and decision-making (Shi et al., 2023). It reduces latency, lowers bandwidth costs, enhances security by processing data locally, and improves system reliability and scalability (Wijesinghe and Kariyawasam, 2024). In space applications, edge devices process data on space stations or lunar bases, enabling immediate environmental adjustments without relying on Earth-based transmission (Dudukovich et al., 2022). In precision agriculture, edge computing supports drones and sensors for real-time plant health and soil monitoring, optimizing resource use and management (Lee et al., 2024).

### 8.2 Standardizing MMA methods

Standardizing MMA methods remains challenging due to many interdependent factors. Environmental variations, including temperature, pressure, light, and humidity, directly influence stress responses, requiring adaptable analytical approaches (Bocklitz et al., 2020). Hardware discrepancies in accuracy and robustness introduce variability, making standardization difficult (Pérez-Patricio et al., 2024). Researchers select analytical and data reduction techniques, such as principal component analysis and eigenvector methods, based on application-specific needs, complicating uniform standardization (Olivieri et al., 2006). MMA integrates multiple bands, modes, and indices, requiring flexible frameworks to handle complex datasets. Application-specific spectral signatures further complicate standardization—normalizing melanin absorption in tissues differs from analyzing scattering effects in plants for early stress detection. Mapping and pattern recognition techniques vary across applications, demanding customized analytical models (Pérez-Patricio et al., 2024). Cross-mode variable contributions shift based on medium properties and sample populations, requiring unique calibration strategies (Bocklitz et al., 2020). Hardware and software components, including device calibration and firmware, dictate sensitivity and responsivity, further preventing universal standards. Image processing techniques rely on specific hardware and software configurations, necessitating tailored filtering, masking, and normalization for each application. While general calibration protocols, such as white or dark normalization, noise reduction, and baseline correction, apply broadly, MMA requires flexible, context-specific protocols (Ajuzieogu, 2017). to reproduce consistent results with field data.

## 9 Findings and results

This review identifies multi-mode analytics as a transformative approach to plant stress assessment, integrating hyperspectroscopy, time-frequency analysis, and advanced techniques for precise detection. Unlike single-mode methods, multi-mode analytics maps stress patterns across spectral bands, enabling earlier and more accurate identification of stress and its root causes. Predicting plant stress remains complex due to spatial, spectral, and physiological interactions. While farmers rely on visual cues like wilting, multi-mode analytics scientifically detects early stress by combining optical, structural, and temporal data.

—This review highlights several key insights that address the questions posed at the outset. Conventional single-mode spectroscopy often fails to capture the multifaceted nature of plant stress by concentrating primarily on isolated indicators, such as reflectance or temperature alone. For example, single-mode approaches may not simultaneously detect complex stress interactions involving drought, salinity, and temperature, as each stressor affects different biochemical pathways. In contrast, the integrated approach of multi-mode analytics significantly enhances accuracy in stress detection by allowing simultaneous assessment of multiple physiological parameters. This multifaceted approach provides a comprehensive overview of plant responses, thus addressing the limitations of single-mode techniques (**Table 9**). ML and AI training begins with establishing ground truth—in this case, tested and verified mappings and patterns to identify plant health anomalies and stress events or pathways. This foundation can accurately predict stress and potential root causes using multiple indices from different spectral bands and detection modalities (Gómez-Chova et al., 2015).

**Table 9:** Limitations of single mode analytics for plant stress assessment.

Parameter	Limitation	Reference
Integration of Stressors	Single-mode spectroscopy often fails to assess multiple stressors simultaneously, leading to an incomplete understanding of plant health.	Atkinson and Urwin, 2012
Focus on Isolated Symptoms	Typically, it concentrates on isolated symptoms rather than providing a comprehensive understanding of underlying stress factors.	Zarco-Tejada et al., 2012
Time-Consuming	Single-mode methods are time-consuming, requiring extensive sample preparation and manual calibration.	AlSuwaidi et al., 2018
Sensitivity to Variability	Single-mode analytics needs to effectively account for environmental variability, leading to inaccuracies in stress detection.	Rahimikhoob et al., 2024
Inability to Capture Dynamics	Lacks the capability to monitor dynamic changes in plant stress conditions effectively since dynamic changes require more than one variable	Emami and Martínez-Muñoz, 2023
Holistic Assessment	It provides data on specific spectral regions but needs a holistic plant health assessment.	Wang et al., 2022
Calibration Challenges	Requires complex calibration processes to ensure accuracy in differing environmental contexts.	Meyer, 2023
Limited Indicators	Concentrating on a few physical or chemical indicators may miss critical metabolites and stress-related physiological responses.	Jorge et al., 2016
Data Interpretation	Requires specialized knowledge for data interpretation, presenting barriers for growers and practitioners with limited technical expertise.	Sarfraz et al., 2023
Maintenance Requirements	It Requires ongoing maintenance and calibration, posing additional operational challenges.	Liu et al., 2019
Spatial Limitations	Single-mode systems often need more spatial resolution to evaluate plant canopy structure effectively.	Maimaitijiang et al., 2020
Temporal Resolution	It provides limited temporal data, hindering the ability to monitor changes over time effectively (the frequency at which data is collected over time).	Atluri et al., 2018
Environmental Sensitivity	Single-mode instruments may be prone to interference from ambient light conditions, affecting measurement accuracy.	Bartula, 2009
Specificity	Low specificity in identifying stressors, sometimes leading to false positives or negatives in stress assessment.	Martinelli et al., 2015
Sample Homogeneity	Assumes sample homogeneity, potentially skewing results if variations exist within the sample.	Ferrari et al., 2004

In response to the advantages of multi-mode spectroscopy (**Table 10**), this review details how combining techniques like HRI, HFI, and Light LiDAR allows for a more thorough examination of plant physiological responses. Each mode has distinct advantages: HRI reveals detailed spectral changes, HFI detects early shifts in photosynthetic activity, and LiDAR captures structural data like leaf orientation. Integrating these methods improves the detection of subtle health indicators, such as chlorophyll content changes or metabolite fluctuations, enabling the early identification of stress that might otherwise go unnoticed.

**Table 10:** Multi-mode analytics methods in plant stress assessment.

Method	Description	Reference
HRI	Providing insights into plant health, including pigment status, water content, and cellular structures. Reflectance mode can be part of any spectral band.	Maimaitijiang et al., 2020
HFI	It focuses on the fluorescence emitted by chlorophyll and other pigments, enabling the detection of changes in photosynthetic activity and early stress indicators.	Pérez-Bueno et al., 2019
LIDAR	It uses laser pulses to measure distances to plant structures, evaluating canopy architecture, leaf orientation, and stress-related structural changes.	Chen, 2013
Thermal Infrared Imaging	Measures thermal emissions from plants to assess leaf and canopy temperatures, offering insights into water stress and transpiration efficiency.	Farella et al., 2022
Data Fusion	It integrates data from multiple sources (e.g., spectral, spatial) to provide a comprehensive view of plant health and improve stress detection accuracy.	Walsh et al., 2024
Machine Learning	Applies algorithms to analyze complex datasets, enhancing the predictive capabilities for identifying stress and developing intervention strategies.	Gómez-Chova et al., 2015
Microstate Analysis	It focuses on a plant’s detailed physiological states, allowing for the identification of early stress signs through the assessment of multiple indicators.	Tomkins et al., 2021
Statistical Methods	Utilizes various statistical techniques to analyze spectral data and extract meaningful patterns related to plant stress conditions.	Nayak et al., 2018
RARS	RARS algorithm uses ratio spectra to establish *linear relationships* between absorption bands and pigment concentrations and determines the contribution of chlorophyll a,b, and carotenoid in reflectance spectra	Chappelle et al, 1992; Kim et al., 1993
Dimensionality Reduction	Utilizes eigenvalues and eigenvectors from PCA to analyze complex datasets, helping to identify patterns that reveal the root causes of plant stress, thereby informing targeted interventions.	Nayak et al., 2018

The review further addresses how advanced analytical methods can enhance predictive capabilities in stress detection. ML and data fusion are valuable in processing complex datasets from multimodal systems, uncovering patterns and correlations that inform predictions about stress causes. For instance, ML algorithms can link FTIR spectroscopy data to physiological stress, offering more profound insights into how molecular changes correlate with stress responses.

Finally, integrating time and frequency domain analysis allows models to capture dynamic, cyclical stress patterns impacting plant health, such as temperature or irrigation cycles. These advanced analytical techniques contribute to a real-time, adaptive approach that improves timely interventions. Based on these findings, the review recommends a coordinated effort toward developing precision multimodal devices to bridge current research gaps, emphasizing that integrated analytical techniques are essential for advancing sustainable agricultural practices in terrestrial and space environments.

### 9.1 Enhanced monitoring

Integrating MMA datasets with sophisticated analytical techniques (Kour et al., 2024) enhances precision farming. These methods promote agricultural sustainability, especially in long-duration space missions, as they significantly mitigate the risks of crop failure. Technologies like hyperspectral imaging, ML, and advanced sensors promote precise crop health monitoring, allowing early detection of stress and nutrient deficiencies for timely interventions (Gao et al., 2020). This level of precision enhances resource efficiency, which is essential for sustainability in space.

Based on these findings, the review recommends the adoption of multimodal analytics (**Table 10**) for devices that integrate different sensing spectral bands and modalities. This approach will bridge research gaps and promote sustainable agricultural practices by enhancing the understanding of plants’ intricate chemical and physiological responses to stressors. Overall, the findings reinforce the transition from traditional single-mode methods towards a more holistic, multimodal strategy that adequately addresses the complexities of plant health challenges.

### 9.2 Challenges in multi-mode analytics

Multi-mode analytics (MMA) provides a cost-effective alternative to expensive methods like X-ray or Raman spectroscopy for plant stress analysis. These systems lower operational costs and simplify technology integration (Meyer, 2023). However, implementation challenges persist, particularly in sensor calibration, cost, and data processing. High-resolution data can cause information overload, complicating actionable insights (Hilbert, 2016). Additionally, maintaining and calibrating sensitive sensors requires technical expertise, which is often scarce in agricultural regions, adding to operational costs (Liu et al., 2019). The affordability of MMA remains a barrier for small-scale farmers, who may lack access to advanced software and trained personnel. Furthermore, data processing demands significant computational resources, potentially limiting adoption in regions with inadequate infrastructure. Environmental variability also affects measurement accuracy, necessitating frequent recalibrations and adaptive models to maintain reliability. To succeed in high-output and small-scale farming, MMA must address cost-effectiveness, ease of use, and scalable data solutions.

### 9.3 Future works

MMA ensure crop viability in extraterrestrial and terrestrial settings, where meticulous resource management becomes essential (Maity and Saxena, 2024). During prolonged space missions, space crews can effectively manage water and nutrient cycles to sustain life and crop productivity. The capability to foresee and mitigate stress factors—such as water shortages, nutrient deficiencies, and environmental challenges—directly contributes to protecting plant health and fulfilling the overarching objectives of long-term human space exploration and habitation. The demand for -devices integrating HFI, HRI, LiDAR, thermal imaging, ML/AI, data Fusion, IBP, time and frequency domain, eigenvectors, and microstate analysis is surging in agricultural (vertical and horizontal farming, horticulture, micro crop plantation) and food industry applications. Multimodal analytics is a Meta-framework, which is pivotal for assessing meat freshness, detecting adulteration in poultry and fish, verifying the purity of olive oil, and identifying pathogens in various food products (Artavia et al., 2021). Their capability for precise, non-destructive testing delivers crucial data that greatly enhances product safety, quality control, and traceability (Vasefi et al., 2018). In terrestrial and space agriculture, these tools are indispensable for monitoring plant health and detecting early signs of disease, sustaining crop yield and quality. In the food sector, they can authenticate product contents, spot adulterants, ensure compliance with safety standards, safeguard consumer health, and support ethical trade practices (Li et al., 2019).

## Data Availability

Publicly available datasets were analyzed in this study. This data can be found here: NA.
